# Equivalence of Alcohol Use Disorder Symptom Assessments in Routine Clinical Care When Completed Remotely via Online Patient Portals Versus In Clinic via Paper Questionnaires: Psychometric Evaluation

**DOI:** 10.2196/52101

**Published:** 2024-07-22

**Authors:** Theresa E Matson, Amy K Lee, Malia Oliver, Katharine A Bradley, Kevin A Hallgren

**Affiliations:** 1 Kaiser Permanente Washington Health Research Institute Seattle, WA United States; 2 Department of Health Systems and Population Health University of Washington Seattle, WA United States; 3 Mental Health and Wellness Department Kaiser Permanente Washington Seattle, WA United States; 4 Department of Medicine School of Medicine University of Washington Seattle, WA United States; 5 Department of Psychiatry and Behavioral Sciences School of Medicine University of Washington Seattle, WA United States

**Keywords:** alcohol, alcohol use disorder, assessment, symptom checklist, electronic health record, patient portal, item response theory, differential item functioning, alcohol use, patient portals, in-clinic, psychometric evaluation, alcoholism, cross-sectional, United States

## Abstract

**Background:**

The National Institute on Alcohol Abuse and Alcoholism (NIAAA) recommends the paper-based or computerized Alcohol Symptom Checklist to assess alcohol use disorder (AUD) symptoms in routine care when patients report high-risk drinking. However, it is unknown whether Alcohol Symptom Checklist response characteristics differ when it is administered online (eg, remotely via an online electronic health record [EHR] patient portal before an appointment) versus in clinic (eg, on paper after appointment check-in).

**Objective:**

This study evaluated the psychometric performance of the Alcohol Symptom Checklist when completed online versus in clinic during routine clinical care.

**Methods:**

This cross-sectional, psychometric study obtained EHR data from the Alcohol Symptom Checklist completed by adult patients from an integrated health system in Washington state. The sample included patients who had a primary care visit in 2021 at 1 of 32 primary care practices, were due for annual behavioral health screening, and reported high-risk drinking on the behavioral health screen (Alcohol Use Disorder Identification Test–Consumption score ≥7). After screening, patients with high-risk drinking were typically asked to complete the Alcohol Symptom Checklist—an 11-item questionnaire on which patients self-report whether they had experienced each of the 11 AUD criteria listed in the *Diagnostic and Statistical Manual of Mental Disorders, Fifth Edition* (*DSM-5*) over a past-year timeframe. Patients could complete the Alcohol Symptom Checklist online (eg, on a computer, smartphone, or tablet from any location) or in clinic (eg, on paper as part of the rooming process at clinical appointments). We examined sample and measurement characteristics and conducted differential item functioning analyses using item response theory to examine measurement consistency across these 2 assessment modalities.

**Results:**

Among 3243 patients meeting eligibility criteria for this secondary analysis (2313/3243, 71% male; 2271/3243, 70% White; and 2014/3243, 62% non-Hispanic), 1640 (51%) completed the Alcohol Symptom Checklist online while 1603 (49%) completed it in clinic. Approximately 46% (752/1640) and 48% (764/1603) reported ≥2 AUD criteria (the threshold for AUD diagnosis) online and in clinic (*P*=.37), respectively. A small degree of differential item functioning was observed for 4 of 11 items. This differential item functioning produced only minimal impact on total scores used clinically to assess AUD severity, affecting total criteria count by a maximum of 0.13 criteria (on a scale ranging from 0 to 11).

**Conclusions:**

Completing the Alcohol Symptom Checklist online, typically prior to patient check-in, performed similarly to an in-clinic modality typically administered on paper by a medical assistant at the time of the appointment. Findings have implications for using online AUD symptom assessments to streamline workflows, reduce staff burden, reduce stigma, and potentially assess patients who do not receive in-person care. Whether modality of *DSM-5* assessment of AUD differentially impacts treatment is unknown.

## Introduction

Nearly 14% of adults in the United States meet criteria for alcohol use disorder (AUD) [1]. However, AUD is underdiagnosed in medical settings [2,3], which decreases opportunities for treatment. Brief screening for unhealthy alcohol use is recommended in primary care, but follow-up assessment of AUD symptoms is necessary to accurately diagnose AUD [4].

The NIAAA recommends brief population-based screening (eg, Alcohol Use Disorder Identification Test–Consumption [AUDIT-C]) and follow-up assessment using the Alcohol Symptom Checklist for patients with high-risk drinking [5]. Previous studies support the validity [6], reliability [7], and clinical utility [8] of the Alcohol Symptom Checklist administered in clinic as part of routine care. However, with increasing virtual care, it is important to test whether measurement characteristics differ across modes of administration. This study builds upon prior psychometric evaluations of the Alcohol Symptom Checklist [6,7] to evaluate whether it had similar measurement characteristics when completed online versus in clinic as part of routine clinical care.

## Methods

### Setting, Design, and Sample

This cross-sectional study used secondary clinical data from electronic health records (EHRs) capturing the Alcohol Symptom Checklist as it was completed during routine care at Kaiser Permanente Washington (KPWA), an integrated health care system in Washington state. KPWA conducts annual behavioral health screening for alcohol, drug use, and depression in primary care [8,9]. Clinical workflows at KPWA for population-based screening and assessment typically occur as follows, although variation in clinical practice is expected: All patients who have not completed screening in the past year are sent a behavioral health screener through the health system’s online patient portal before scheduled primary care appointments as part of electronic check-in. If high-risk drinking is reported during online screening, the Alcohol Symptom Checklist automatically displays in the portal (with exceptions; Multimedia Appendix 1). Patients who do not complete screening online are typically prompted by check-in staff or medical assistants (MAs) to complete screening in clinic on paper during primary care appointments, with results entered into EHRs by MAs. If high-risk drinking is reported in clinic, the EHR prompts MAs to give patients a paper-based version of the Alcohol Symptom Checklist to complete in clinic [9]. Patients complete in-clinic screening and assessment for several reasons: some may not have set up their online patient portal, others may need translated versions in a language other than English, while others choose not to complete the checklist online. During the study period, approximately 90% of all patients with a primary care appointment completed alcohol screening and approximately 80% of primary care patients reporting high-risk drinking completed the follow-up Alcohol Symptom Checklist.

Adult patients (aged ≥18 years) were included in this study if they completed a primary care appointment (phone, video, office) during 2021 and were due for annual screening, reported high-risk drinking on the alcohol screening measure (AUDIT-C score ≥7 [10-13]; Multimedia Appendix 2), and completed the Alcohol Symptom Checklist. Checklists with missing items were excluded (Multimedia Appendix 1). If patients completed ≥1 checklist in clinical care, a single checklist was randomly selected for analysis.

### Ethical Considerations

This deidentified data-only study was approved by the KPWA Health Research Institute Institutional Review Board (reference 1481289-1) with waivers of consent and Health Insurance Portability and Accountability Act authorization.

### Measures

The Alcohol Symptom Checklist is a valid [6] and reliable [7] 11-item questionnaire assessing AUD criteria over a past-year timeframe, mirroring the *Diagnostic and Statistical Manual of Mental Disorders, Fifth Edition* (*DSM-5*; Multimedia Appendix 3) [14]. Patients self-reported whether they experienced each of 11 *DSM-5* AUD criteria (yes/no). Summed scores ranged from 0 to 11, reflecting the number of AUD criteria endorsed and informing the severity of AUD (per the *DSM-5*, mild=2-3, moderate=4-5, and severe=6-11 criteria) [14].

Modality of administration was coded as online or in clinic (Multimedia Appendix 1).

EHR-documented demographics included age, sex, race, ethnicity, and need for a language interpreter.

### Analyses

Descriptive statistics characterized the demographics of patients completing online and in-clinic checklists. Logistic regression models estimated the prevalence of each AUD criterion and of symptoms consistent with mild, moderate, or severe AUD across modalities, adjusted for demographic characteristics that significantly differed between groups.

Main analyses used item response theory [15] to evaluate differential item functioning (DIF) on checklist items across modalities (Multimedia Appendix 4 [6,14-20]).

DIF can be statistically significant without having a clinically meaningful impact on determination of AUD severity [15]. Clinicians typically make diagnostic and treatment decisions based on total scores rather than individual items because the number of criteria informs whether AUD is likely present (≥2 criteria) and its severity [14]. We evaluated the cumulative impact of DIF by estimating how total scores would be expected to change due to DIF if a patient with the same level of latent AUD completed checklists online vs in clinic (Multimedia Appendix 4 [6,14-20]).

## Results

Among 3243 patients who completed the Alcohol Symptom Checklist, 1640 (51%) completed the checklist online, while 1603 (49%) completed the checklist in clinic. Among those excluded were 1 online and 63 in-clinic checklists that were missing items (Multimedia Appendix 1). Patients were predominantly middle-aged, male, White, and non-Hispanic. There were small but significant differences across modalities, with more diversity observed among those who completed checklists in clinic (Table 1).

After adjustment for demographic characteristics, item endorsement ranged from 12.4% (hazardous use) to 38.5% (physical or psychological problems) on checklists completed online and 8.3% (hazardous use) to 39.4% (larger and longer) on checklists completed in clinic. Item endorsement differed significantly by modality for 5 items (Table 2). However, prevalence of AUD and AUD severity levels did not differ by modality (*P*=.37 and *P*=.39, respectively).

**Figure 1:**
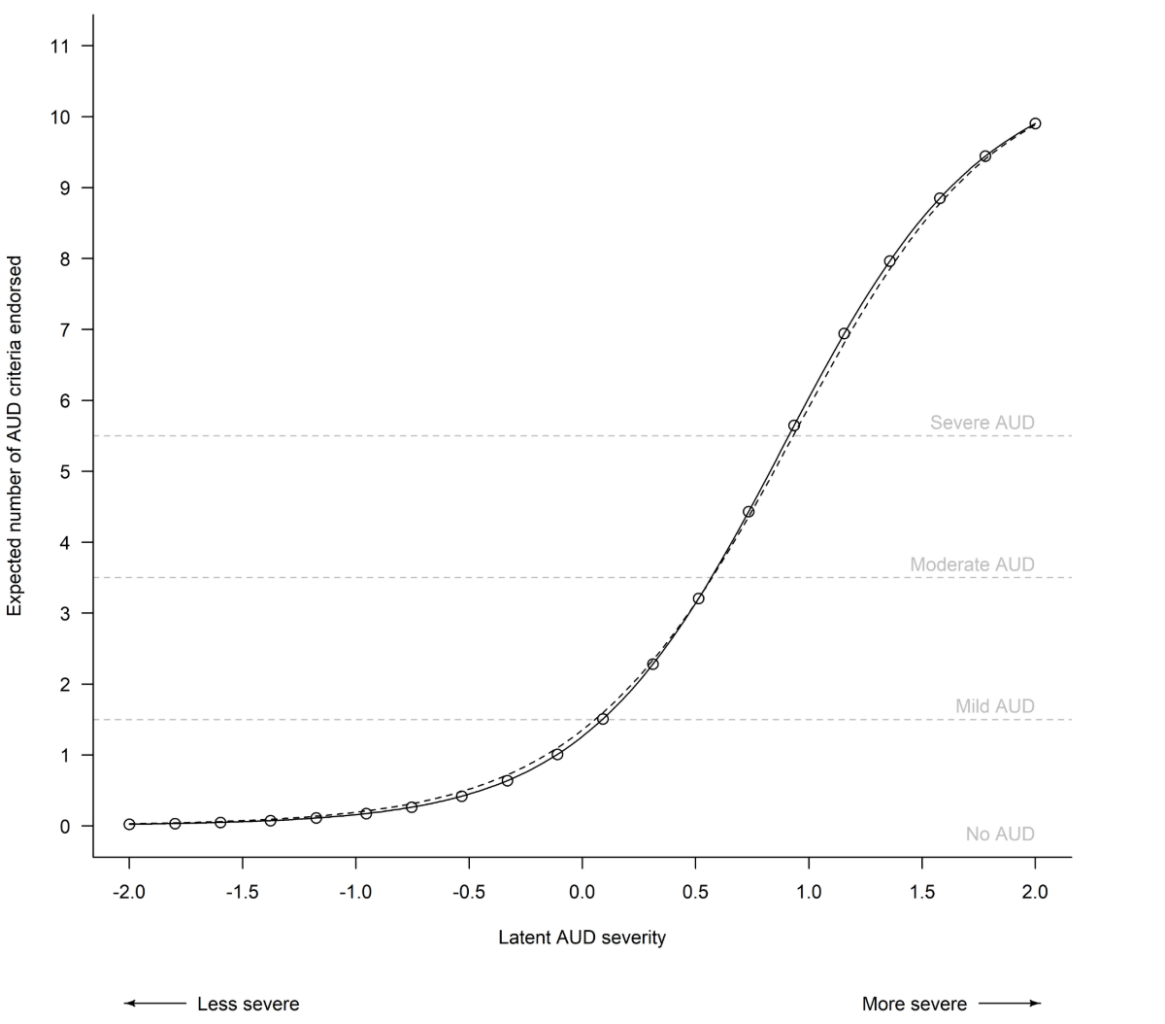
Item response theory analysis results displaying the expected number of alcohol use disorder (AUD) criteria endorsed on the Alcohol Symptom Checklist (y-axis) for each assessment modality (online versus in-clinic; separate lines) based on an individual’s latent AUD severity (x-axis). Vertical distances between the plotted lines indicate the expected differences in the number of AUD criteria endorsed for a person with the same level of latent AUD severity who completed the Alcohol Symptom Checklist online vs in person. Note that the lines nearly overlap, which is reflective of the small cumulative impact of differential item functioning between the 2 assessment modalities on expected Alcohol Symptom Checklist total scores (Multimedia Appendix 4 [6,14-20] provides additional analytic details).

DIF for online versus in-clinic modalities was observed for 4 items (Table 3). However, this had minimal impact on total scores: for patients with equal levels of latent AUD severity, estimated differences in the number of AUD criteria endorsed were expected to differ by <0.13 criteria (out of 11) due to DIF (Figure 1). In other words, DIF did not substantially impact criteria counts used by clinicians for diagnosing.

**Table 1:** Patient characteristics overall and across modality of Alcohol Symptom Checklist administration.

Patient characteristics			Total (N=3243), n (%)		In clinic (n=1603), n (%)		Online portal (n=1640), n (%)		*P* value	
**Age (years)**										.001
	18-24	254 (7.8)		148 (9.2)		106 (6.5)				
	25-44	1528 (47.1)		707 (44.1)		821 (50.1)				
	45-64	1152 (35.5)		596 (37.2)		556 (33.9)				
	≥65	309 (9.5)		152 (9.5)		157 (9.6)				
**Sex**										.36
	Female	930 (28.7)		448 (27.9)		482 (29.4)				
	Male	2313 (71.3)		1155 (72.1)		1158 (70.6)				
**Race**										<.001
	American Indian/Alaska Native	64 (2)		30 (1.9)		34 (2.1)				
	Asian	193 (6)		89 (5.6)		104 (6.3)				
	Black	151 (4.7)		100 (6.2)		51 (3.1)				
	Native Hawaiian/Pacific Islander	53 (1.6)		30 (1.9)		23 (1.4)				
	White	2271 (70)		1104 (68.9)		1167 (71.2)				
	Other	88 (2.7)		58 (3.6)		30 (1.8)				
	Unknown	423 (13)		192 (12)		231 (14.1)				
**Ethnicity**										.01
	Hispanic	209 (6.4)		124 (7.7)		85 (5.2)				
	Not Hispanic	2014 (62.1)		977 (60.9)		1037 (63.2)				
	Unknown	1020 (31.5)		502 (31.3)		518 (31.6)				
**Needs interpreter**										<.001
	Yes	40 (0.3)		35 (2.2)		5 (0.3)				
	No	86 (2.7)		47 (2.9)		39 (2.4)				
	Unknown	2117 (96.1)		1521 (94.9)		1596 (97.3)				

**Table 2:** Prevalence of Alcohol Symptom Checklist item endorsement across modality of administration, adjusted for demographic characteristics that differed between groups^a^.

		In-clinic (n=1603), n (%; 95% CI)	Online portal (n=1640), n (%; 95% CI)	*P* value	
**Alcohol Symptom Checklist items**					
	1. Tolerance	413 (25.8; 23.7-28.0)	464 (28.2; 26.1-30.4)	.12	
	2. Withdrawal	245 (15.4; 13.6-17.1)	258 (15.7; 13.9-17.4)	.82	
	3. Larger/longer	547 (34.1; 31.8-36.4)	647 (39.4; 37.1-41.8)	.002^b^	
	4. Quit/control	428 (26.6; 24.4-28.7)	348 (21.3; 19.3-23.3)	<.001^b^	
	5. Time spent	341 (21.4; 19.4-23.4)	394 (23.9; 21.9-25.9)	.09	
	6. Physical/psychological problems	613 (38.5; 36.1-40.9)	502 (30.4; 28.2-32.6)	<.001^b^	
	7. Neglect roles	202 (12.6; 10.9-14.2)	185 (11.3; 9.8-12.9)	.28	
	8. Hazardous use	200 (12.4; 10.8-14.0)	135 (8.3; 6.9- 9.6)	<.001^b^	
	9. Social/interpersonal problems	383 (23.6; 21.5-25.7)	273 (16.9; 15.1-18.7)	<.001^b^	
	10. Craving	463 (29.0; 26.8-31.3)	439 (26.6; 24.5-28.8)	.13	
	11. Activities given up	284 (17.8; 15.9-19.7)	278 (16.9; 15.1-18.7)	.52	
**AUD^c^ severity based on criteria endorsed**					.39
	No AUD (0-1 criteria)	839 (52.3; 49.9-54.8)	888 (54.1; 51.7-56.5)		
	Mild AUD (2-3 criteria)	302 (18.8; 16.9-20.8)	310 (18.9; 17.0-20.8)		
	Moderate AUD (4-5 criteria)	176 (10.9; 9.4-12.5)	185 (11.3; 9.8-12.9)		
	Severe AUD (≥6 criteria)	286 (17.9; 16.0-19.8)	257 (15.6; 13.9-17.4)		
Number of AUD criteria^d^, mean (95% CI)		2.6 (2.4-2.7)	2.4 (2.3-2.5)	.08	

^a^Adjusted for patient age, race, ethnicity, and need for an interpreter.

^b^Significant at α level of .05.

^c^AUD: alcohol use disorder.

^d^Estimated using adjusted linear regression.

**Table 3:** Differential item functioning across modality of Alcohol Symptom Checklist administration. Discrimination parameters *a* characterize how well each item differentiates higher versus lower alcohol use disorder (AUD) severity, and severity parameters *b* characterize where, along the continuum of latent AUD severity, the item best discriminates (Multimedia Appendix 4 [6,14-20] provides additional analytic details). For the full test parameters, the latent mean and latent variance in clinic were 0.00 and 1.00, while for the online portal the values were –0.05 and 0.90. Latent means and variances were fixed to 0 and 1, respectively, for the reference group and were freely estimated for nonreference groups.

Alcohol Symptom Checklist item	In clinic^a^		Online portal^b^	
	*a*	*b*	*a*	*b*
1. Tolerance	1.46	0.99	—^c^	0.81
2. Withdrawal	2.19	1.25	—	—
3. Larger/longer	2.18	0.52	—	0.28
4. Quit/control	2.68	0.73	—	0.87
5. Time spent^d^	2.8	0.84	—	—
6. Physical/psychological problems^d^	2.74	0.44	—	—
7. Neglect roles^d^	3.26	1.29	—	—
8. Hazardous use	1.54	1.86	—	—
9. Social/interpersonal problems	3.08	0.81	2.65	1.07
10. Craving	2.51	0.68	—	—
11. Activities given up	3.51	1	—	—

^a^Indicates reference group.

^b^Item parameters that significantly differed (α=.0045) from the reference group are presented in the table.

^c^Item parameters that did not significantly differ from the reference group were fixed to equality to improve the power of significance testing for remaining items. These and items that were fixed as anchoring items are indicated with dashes (—).

^d^Indicates anchor item.

## Discussion

### Principal Findings

The Alcohol Symptom Checklist administered online performed similarly to the checklist completed in clinic. Small differences in the prevalence of item endorsement (<8%) did not result in differential AUD or AUD severity across modalities. While DIF across modalities was identified for 4 items, it did not meaningfully impact total scores, which are used by clinicians to diagnose AUD. The lack of clinically meaningful impact of DIF across modalities supports the use of online portals or in-clinic paper forms for administering the Alcohol Symptom Checklist in primary care to facilitate accurate AUD diagnosis.

Our findings are consistent with prior work suggesting pragmatic symptom-based checklists have high test-retest reliability [7] and perform similarly across patient demographic characteristics [6,20] and treatment status [21]. Our study adds to the growing body of evidence supporting the validity and reliability of the Alcohol Symptom Checklist completed in real-world routine care settings.

Our findings have important implications for implementing assessment in health care. In-clinic administration may be preferable for clinics that lack online resources (eg, web developers) or online infrastructure (eg, patient portals) to support integrating questionnaires into EHRs and remote workflows. When these resources are available, online administration may be preferable to help streamline workflows, reduce staff burden, and ensure more complete responses (eg, fewer missed items).

Both assessment modalities allow patients to directly report AUD symptoms (rather than staff asking the questions), which decreases stigma and improves screening quality, screening feasibility, and patient comfort [22]. Assessment of AUD symptoms remotely via an online portal may further reduce stigma and increase comfort in self-reporting alcohol-related concerns [23].

The heterogeneity of patients who completed the Alcohol Symptom Checklist online versus in clinic is an important finding that underscores the need for both modalities to avoid inequitable identification of AUD. Young adults (aged 18-24 years), older adults (aged ≥65 years), patients of minoritized racial and ethnic backgrounds, and patients who needed an interpreter were more likely to complete checklists in clinic. As digital tools become increasingly common in health care [24], offering both modalities may help minimize inequities. For example, online portals may be more accessible to patients who do not access in-person services (eg, due to logistical, financial, health status, or other factors), while the in-clinic modality may have greater reach for patients without reliable internet or digital devices, who have lower digital literacy, who need translated materials, who do not use patient portals, and who may be more comfortable responding when they can ask questions or explain responses to a health care provider [25].

### Limitations

This study has limitations. Patients may have underestimated or underreported their alcohol use and AUD symptoms. Racial and ethnic diversity were limited. The sample included patients who completed annual screening at a primary care appointment and thus represent the population that uses care but may not represent other groups that do not receive primary care. Results may not generalize to other populations or health systems. The study has noteworthy strengths. While processes for completing the Alcohol Symptom Checklist online versus in clinic differed, these differences reflect actual clinical assessment procedures, providing high external validity regarding how checklists perform under real-world conditions. The sample size was large and powered to detect small amounts of measurement invariance. Future studies should assess whether modality of AUD assessment differentially impacts diagnosis and treatment.

### Conclusion

Remote completion of the Alcohol Symptom Checklist online by primary care patients performed similarly to a valid [6] and reliable [7] paper-based version completed in clinic. Future studies should evaluate methods to support health care providers in following and treating patients with AUD through in-person and virtual care.

## Appendix

Multimedia Appendix 1 Study sample selection.

Multimedia Appendix 2 Alcohol Use Disorders Identification Test-Consumption (AUDIT-C).

Multimedia Appendix 3 Alcohol Symptom Checklist.

Multimedia Appendix 4 Additional analytical details.

## Abbreviations

AUD: alcohol use disorder
AUDIT-C: Alcohol Use Disorder Identification Test–Consumption
DIF: differential item functioning
*DSM-5*: *Diagnostic and Statistical Manual of Mental Disorders, Fifth Edition*
EHR: electronic health record
KPWA: Kaiser Permanente Washington
MA: medical assistant
NIAAA: National Institute on Alcohol Abuse and Alcoholism
