# Supplementary material for: Equivalence of Alcohol Use Disorder Symptom Assessments in Routine Clinical Care When Completed Remotely via Online Patient Portals Versus In Clinic via Paper Questionnaires: Psychometric Evaluation
Source: J Med Internet Res. 2024 Jul 22;26:e52101. doi: 10.2196/52101 (PMC11301125; doi:10.2196/52101)
Supplement: Multimedia Appendix 1 [file jmir_v26i1e52101_app1.docx]

**Supplement 1:** Study sample selection

**Supplement 2 caption:**

^a^ The electronic health record will automatically ask patients to complete annual alcohol screening and/or remind in-clinic staff to administer alcohol screens to patients if patients have not completed screening in the year prior.

^b^ There are several reasons patients may not have completed the Alcohol Symptom Checklist despite reporting high-risk drinking. First, patients who screened positive for depression and had suicidal thoughts were given a suicide risk assessment instead. Second, on May 30, 2021, the electronic health record prompts that automatically displayed the Alcohol Symptom Checklist after patients reported a high-risk alcohol use (AUDIT-C scores ≥7), were inadvertently disabled. However, a web-based version of the Alcohol Symptom Checklist may still have displayed through the online patient portal if patients had previously reported high-risk alcohol use or patients may have provided this information in-clinic. Third, patients may have declined to complete the Alcohol Symptom Checklist.

^c^ Patients could skip any item on the Alcohol Symptom Checklist they did not want to answer. However, most completed all 11 items.

^d^ Virtual appointments were by phone or video

^e^ When completed in clinic, Alcohol Symptom Checklists are typically administered on paper. However, a small subset of patients may have completed the Alcohol Symptom Checklist verbally as part of a virtual visit. Responses to checklist items were recorded in clinic by check-in staff or medical assistants.

64 patients had missing items on the Alcohol-Symptom Checklist^c^ Among them:

- 1 patient completed the checklist online
- 63 patients completed the checklist in clinic

187,276 patients did not report high-risk alcohol use on alcohol screening (AUDIT-C < 7)

1,245 patients did not complete the Alcohol Symptom Checklist^b^

3,243 patients included in analyses

191,828 patients were due for annual alcohol screening as part of routine primary care (i.e., had not completed alcohol screening in the year prior)^a^

604 patients had a virtual appointment^d^

247 patients had a virtual appointment^d,e^

4,552 patients reported high-risk alcohol use on alcohol screening (AUDIT-C ≥ 7)

3,307 patients completed an Alcohol Symptom Checklist

1,640 patients remotely completed the Alcohol Symptom Checklist via online patient portal before a primary care appointment

1,603 patients completed the Alcohol Symptom Checklist in clinic during check-in for a primary care appointment

1,036 patients had an office appointment

1,356 patients had an office appointment
