# Supplementary figures and images for: Equivalence of Alcohol Use Disorder Symptom Assessments in Routine Clinical Care When Completed Remotely via Online Patient Portals Versus In Clinic via Paper Questionnaires: Psychometric Evaluation
Source: J Med Internet Res. 2024 Jul 22;26:e52101. doi: 10.2196/52101 (PMC11301125; doi:10.2196/52101)

**Supplement 2:** Alcohol Use Disorders Identification Test-Consumption (AUDIT-C)


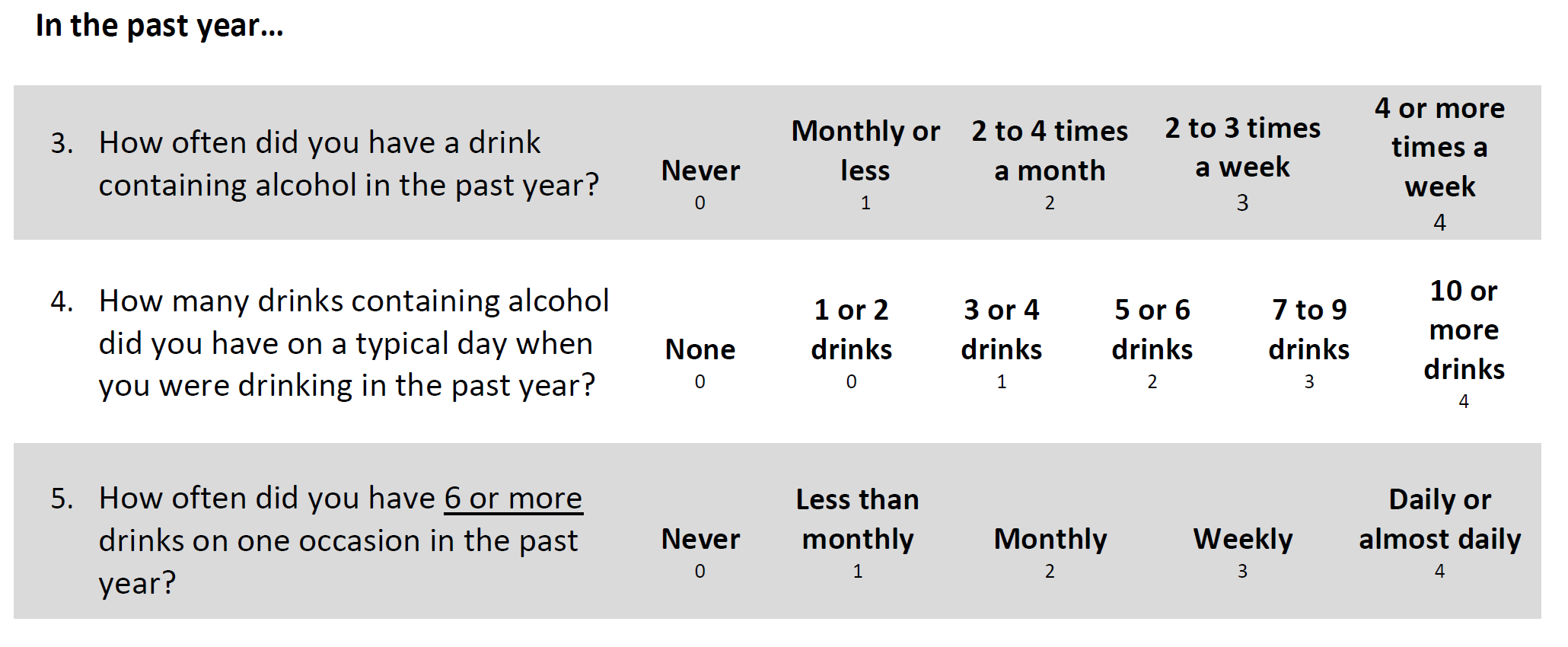

Supplement: Multimedia Appendix 2 [file jmir_v26i1e52101_app2.docx]

**Supplement 3:** Alcohol Symptom Checklist

**
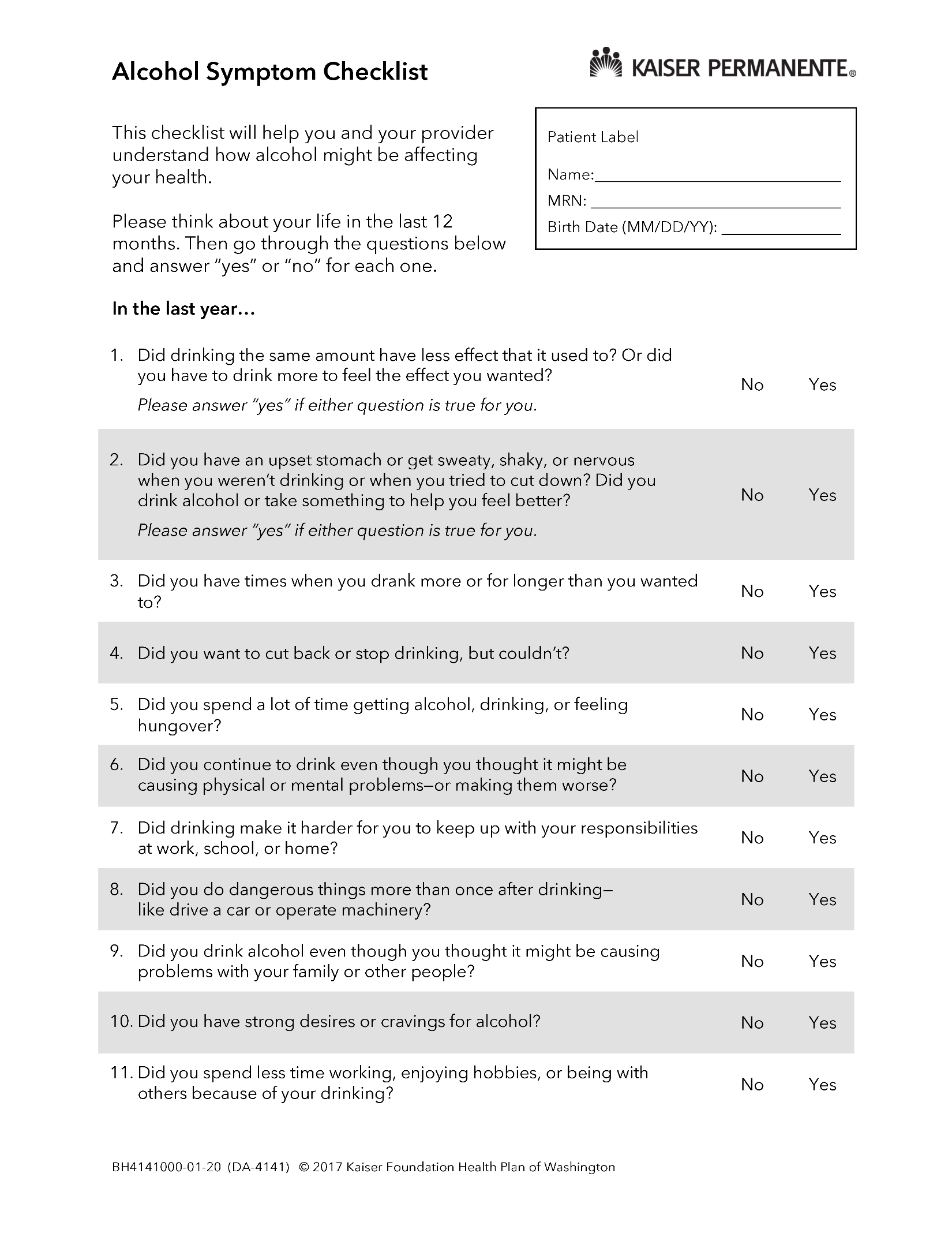
**

Supplement: Multimedia Appendix 3 [file jmir_v26i1e52101_app3.docx]
