# Supplementary material for: Equivalence of Alcohol Use Disorder Symptom Assessments in Routine Clinical Care When Completed Remotely via Online Patient Portals Versus In Clinic via Paper Questionnaires: Psychometric Evaluation
Source: J Med Internet Res. 2024 Jul 22;26:e52101. doi: 10.2196/52101 (PMC11301125; doi:10.2196/52101)
Supplement: Multimedia Appendix 4 [file jmir_v26i1e52101_app4.docx]

**Supplement 4:** Additional analytic detail

We used Item Response Theory (IRT) to characterize and compare psychometric properties of Alcohol Symptom Checklist Items completed online versus in clinic. Similar to a prior validation study [1], the 11 checklist items were modeled as loading onto a single, continuous latent variable reflecting AUD severity using a two-parameter logistic IRT model [2]. Two parameters were estimated for each item: discrimination (*a*) and severity (*b*). Respectively, these parameters characterize how well each item differentiates higher versus lower AUD severity and where, along the continuum of latent AUD severity, the item best discriminates [3, 4]. A series of likelihood ratio tests evaluated whether item discrimination and severity parameters differed significantly across online and in-clinic modalities (described below). Parameters for three “anchor” items (time spent, physical/psychological problems, neglect roles) were fixed to equality across the two modalities to ensure equivalent measurement of latent means and variances [5]. Selection of anchor items was based on a previous study [1].

To test for differential item functioning (DIF), a series of item response theory models (IRT) models were fit and compared using likelihood ratio testing. In the first model, discrimination (*a*) and severity (*b*) parameters for each of the 11 items on the Alcohol Symptom Checklist were fixed to equality between the two in-clinic and online modalities (i.e., a model that assumed no DIF based on modality). In the next model, item #1 was estimated allowing for independent estimation of discrimination and severity parameters for each modality (i.e., a model that allowed potential DIF to be accounted for through different parameters for each modality). A likelihood ratio test was computed to test whether the model that allowed both modalities to have their own independently estimated parameters for item #1 provided significantly better fit than the simpler model that constrained the two groups have identical parameters. When the likelihood ratio test was significant, it was concluded that DIF was present for item #1. When the likelihood ratio test was not significant, it was concluded that DIF was not present for item #1. This process was repeated for each item on the Alcohol Symptom Checklist, and alpha level was adjusted to 0.045 (0.05/11 items) to account for multiple comparisons. Importantly, when a parameter does not significantly differ between groups, it is recommended to keep that parameter fixed to equality (i.e., so the model estimates identical values of that parameter for both groups) because this improves the power of the significance testing for the remaining items that will be tested [2, 5]. Therefore, in a final model, item parameters that significantly differed were independently estimated and item parameters that did not significantly differ in the above steps were fixed to equality along with three “anchor” items (time spent, physical/psychological problems, neglect roles). Keeping the anchor items fixed to equality across the two modalities was necessary to account for potential differences in latent means and variances across the two modalities; failing to account for them could bias DIF tests due to unmodeled differences in latent means and latent variances being recognized as DIF. In sensitivity analyses, we tested whether similar results were found by selecting three alternate anchor items (withdrawal, hazardous use, craving). Sensitivity analyses that selected three alternate anchor items (withdrawal, hazardous use, craving) [6] yielded similar results.

DIF can be present but have minimal impact on total scores (i.e., DIF can be present in very small amounts but statistically significant due to a large sample size, or DIF can be present in opposite directions for different items, effectively cancelling out) [5]. Providers typically don’t make diagnoses or determine severity based on the presence/absence of individual items on the Alcohol Symptom Checklist but rather on the cumulative count of items a patient endorses (i.e., total scores reflecting symptom counts) [7]. For example, the presence of 2 or more AUD criteria is sufficient for AUD diagnosis according to DSM-5, and the number of criteria present is also used to determine AUD severity according to DSM-5 (2-3 reflecting mild AUD, 4-5 reflecting moderate AUD, 6-11 reflecting severe AUD). Therefore, consistent with prior studies [1, 8], we were particularly focused on conducting differential test functioning (DTF) analyses to examine the cumulative impact of DIF on Alcohol Symptom Checklist total scores. We conducted DTF analyses by estimating the expected total scores (i.e., symptom counts) on the Alcohol Symptom Checklist that would be expected for any individual with a given latent AUD severity who completed a checklist online and compared that to the estimated total scores (i.e., symptom counts) that would be expected for the same individual who completed a checklist in clinic. In other words, this difference in expected total scores (online vs. in clinic) represents the difference in expected total scores that would be attributable to the cumulative impact of differential item functioning on all of the Alcohol Symptom Checklist items. A large amount of DTF (e.g., >1 point difference) would indicate that total scores need to be interpreted differently for in-clinic and online modalities (e.g., different cut-offs for diagnosing), because the number of criteria endorsed could potentially be attributable in part to the modality on which the checklist was completed, rather than the level of latent AUD severity. In contrast, a small amount of DTF (e.g., <1 point difference) would indicate that total scores can be interpreted similarly across modalities, because the number of criteria endorsed was not likely attributable to the modality on which the checklist was completed.

**References for Supplement 4**

1. Hallgren, K.A., et al., *Practical Assessment of Alcohol Use Disorder in Routine Primary Care: Performance of an Alcohol Symptom Checklist.* J Gen Intern Med, 2021.

2. Chalmers, R., *mirt: A Multidimensional Item Response Theory Package for the R Environment.* Journal of Statistical Software, 2012. **48**(6): p. 1-29.

3. Nguyen, T.H., et al., *An introduction to item response theory for patient-reported outcome measurement.* Patient, 2014. **7**(1): p. 23-35.

4. Hays, R.D., L.S. Morales, and S.P. Reise, *Item response theory and health outcomes measurement in the 21st century.* Med Care, 2000. **38**(9 Suppl): p. II28-42.

5. Chalmers, R.P., A. Counsell, and D.B. Flora, *It Might Not Make a Big DIF: Improved Differential Test Functioning Statistics That Account for Sampling Variability.* Educ Psychol Meas, 2016. **76**(1): p. 114-140.

6. Kopf, J., A. Zeileis, and C. Strobl, *Anchor Selection Strategies for DIF Analysis: Review, Assessment, and New Approaches.* Educ Psychol Meas, 2015. **75**(1): p. 22-56.

7. American Psychiatric Association, *Diagnostic and statistical manual of mental disorders (5th ed.)*. 2013.

8. Matson, T.E., et al., *Psychometric Performance of a Substance Use Symptom Checklist to Help Clinicians Assess Substance Use Disorder in Primary Care.* JAMA Netw Open, 2023. **6**(5): p. e2316283.
